# Investigating Sterol and Redox Regulation of the Ion Channel Activity of CLIC1 Using Tethered Bilayer Membranes

**DOI:** 10.3390/membranes6040051

**Published:** 2016-12-08

**Authors:** Heba Al Khamici, Khondker R. Hossain, Bruce A. Cornell, Stella M. Valenzuela

**Affiliations:** 1 School of Life Sciences, University of Technology Sydney, Sydney 2007, Australia; heba.alkhamici@uts.edu.au (H.A.K.); khondker.r.hossain@student.uts.edu.au (K.R.H.); 2 Australian Centre for Neutron Scattering, Australian Nuclear Science and Technology Organisation (ANSTO), NSW 2234, Australia; 3 Surgical Diagnostics Pty Ltd., Sydney 2069, Australia; brucec.sdx@gmail.com

**Keywords:** CLIC, chloride intracellular ion channel proteins, tethered lipid membranes, cholesterol, ergosterol

## Abstract

The Chloride Intracellular Ion Channel (CLIC) family consists of six conserved proteins in humans. These are a group of enigmatic proteins, which adopt both a soluble and membrane bound form. CLIC1 was found to be a metamorphic protein, where under specific environmental triggers it adopts more than one stable reversible soluble structural conformation. CLIC1 was found to spontaneously insert into cell membranes and form chloride ion channels. However, factors that control the structural transition of CLIC1 from being an aqueous soluble protein into a membrane bound protein have yet to be adequately described. Using tethered bilayer lipid membranes and electrical impedance spectroscopy system, herein we demonstrate that CLIC1 ion channel activity is dependent on the type and concentration of sterols in bilayer membranes. These findings suggest that membrane sterols play an essential role in CLIC1’s acrobatic switching from a globular soluble form to an integral membrane form, promoting greater ion channel conductance in membranes. What remains unclear is the precise nature of this regulation involving membrane sterols and ultimately determining CLIC1’s membrane structure and function as an ion channel. Furthermore, our impedance spectroscopy results obtained using CLIC1 mutants, suggest that the residue Cys24 is not essential for CLIC1’s ion channel function. However Cys24 does appear important for optimal ion channel activity. We also observe differences in conductance between CLIC1 reduced and oxidized forms when added to our tethered membranes. Therefore, we conclude that both membrane sterols and redox play a role in the ion channel activity of CLIC1.

## 1. Introduction

The Chloride Intracellular Ion Channel (CLIC) family members contain no obvious transmembrane domain in their protein structure; nevertheless, they are capable of inserting into phospholipid membranes directly from their soluble state, where they can function as ion channels [1,2]. This has allowed for their ease of study using a range of artificial lipid membrane systems including tip-dip and tethered bilayer membranes (tBLMs) [3,4]. Reported conductance levels from single channel recordings of the ion channel activity of recombinant CLIC1 (rCLIC1) added to artificial membrane systems and from cells over-expressing rCLIC have been varied [5,6]. For example, single channel conductance recordings of rCLIC1 added to bilayer membranes made from asolectin, phosphatidylethanolamine (PE) and phosphotidylserine (PS) lipids were 60 pS and 120 pS. Its conductance in membranes containing PC lipids was 28 pS [4,7], while single channel conductance levels recorded from CLIC1-transfected CHO-K1 cells were 8 pS and 16 pS [4,7,8,9].

Similar variations in the conductance levels of CLIC4 were also reported following patch clamp measurements. Reported CLIC4 single channel activity was higher from bilayer membrane recordings using reconstituted brain microsomes [10,11], as compared to recordings obtained from adding rCLIC4 to artificial membranes containing neutral lipids only [11,12,13,14]. Therefore, it has been proposed that CLIC protein channel activity is sensitive to membrane lipid composition in addition to previously demonstrated external membrane factors, including redox environment and pH [7,10]. The localization of the protein within membranes was also varied, with CLIC4 found to localize to cholesterol rich micro-domains called caveolae [15,16,17], which led to the proposal that CLIC proteins may only be functional in membranes containing cholesterol. In a study using phospholipid vesicle chloride efflux assays by Tulk et al., 2002 [7], it was found that CLIC1 demonstrated no ion channel activity in membranes containing pure neutral lipid mixtures, while activity was greater in membranes containing 10% of a negatively charged phospholipid such as phosphotidylserine (PS) or phosphatidic acid (PA). Increasing cholesterol concentration to 30 mol % in these membranes caused the channel activity of CLIC1 to be suppressed [7]. Conversely, a study by Singh, et al. [10] using Langmuir-film monolayers and patch clamping techniques, shows that membranes containing POPE, POPS and cholesterol in a molar ratio of 4:1:1 induced CLIC1 membrane insertion and ion channel conductance. However, in both these studies the role of cholesterol in CLIC1’s membrane insertion or its channel conductance activity was not explored. Subsequently, Valenzuela et al., [3] confirmed that CLIC1 ion channel conductance is optimal when inserted into membranes containing cholesterol. This increased conductance can be correlated with an increase in membrane insertion of the protein, as we have previously demonstrated by Langmuir monolayer studies that indicate both sterol and phospholipid membrane composition, regulate CLIC1 membrane insertion [18]. 

Here-in, we further investigate the effect of two different sterols (mammalian sterol, cholesterol and the fungal sterol, ergosterol) on the membrane conductance activity of CLIC1. Membrane sterols are known to affect the stability of ion channels and pore proteins in membranes which is an important factor in maintaining the rate of ion transport across membranes [19]. The effect of these sterols on the ion channel conductance of CLIC1 can indicate a potential role for CLIC1 as an anti-fungal agent, acting in a manner similar to nystatin A and amphotericin B. The approach we have employed in this study is to use tethered bilayer lipid membranes (tBLMs) and Electrical Impedance Spectroscopy (EIS). tBLMs have increasingly become the tools of choice for the study of protein and membrane interactions. This is due to their ease of use, stability and the ability to tailor their lipid composition in order to mimic natural cell membranes [20,21,22]. The influence of the oxidation state of the protein CLIC1, on its conductance activity was also assessed, using monomer (reduced) and dimer (oxidized) versions of the protein.

## 2. Results and Discussion

### 2.1. Conductance Properties of CLIC1 Reduced Monomer and CLIC1 Oxidised Dimer in tBLMs

In order to study the effect of cholesterol on CLIC1’s ion channel function, rCLIC1 (reduced and oxidised forms) were added to sealed tBLMs containing phospholipids and varied concentrations of cholesterol, where the initial conductance of all the freshly formed membranes was stabilised to a baseline value of less than 1 μS and with a capacitance ranging between 20 and 23 nF. Both the conductance and capacitance of the tBLMs were measured using EIS, as previously described [3].

**Figure 1:**
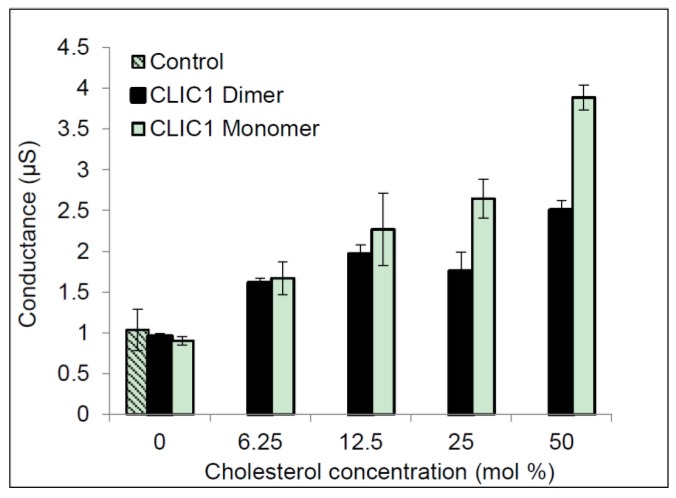
Conductance of CLIC1 in tBLMs containing varying amounts of cholesterol. 20 µg of CLIC1 oxidised dimer was pre-incubated with 2 mM H_2_O_2_; 20 µg of CLIC1 reduced monomer was pre-incubated with 0.5 mM TCEP in 100 µL of HEPES/KCl buffer (pH 6.5) prior to adding to tethered bilayer membranes containing 0, 6.25, 12.5, 25 and 50 mol % cholesterol concentrations. Control is membrane with 0 mol % cholesterol containing 100 µL HEPES/KCl buffer (pH 6.5) with no protein added. The error bars represent the standard error of three independent impedance spectroscopy conductance measurements (*n* = 3).

Figure 1 shows the results for CLIC1 reduced monomer (protein had been purified and subsequently stored in the presence of 0.5 mM TCEP) and CLIC1 dimer (protein purified and subsequently stored in the presence of 2 mM H_2_O_2_). Both showed little to no conductance in membranes containing no cholesterol. Conduction levels were similar to the control, in which no protein was added to membranes containing 50 mol % cholesterol. These data are in agreement with our previous study using monomer rCLIC1 [3]. In membranes containing 6.25 mol % cholesterol, both rCLIC1 monomer and dimer samples showed increases in their conductance. The conductance appears to increase proportionally as the level of cholesterol increased from 12.5 mol % to 25 mol % and was highest in membranes containing 50 mol % cholesterol. ANOVA and regression analysis confirm a significant (*p* value < 0.005) difference exists among all of the tests samples: CLIC1 reduced monomer and CLIC1 oxidised dimer samples in membranes with different cholesterol concentrations, compared to CLIC1 conductance measured in membranes containing no cholesterol (see Figure 1). However, there was no significant difference between the CLIC1 monomer and dimer within membranes containing varying amounts of cholesterol.

**Figure 2:**
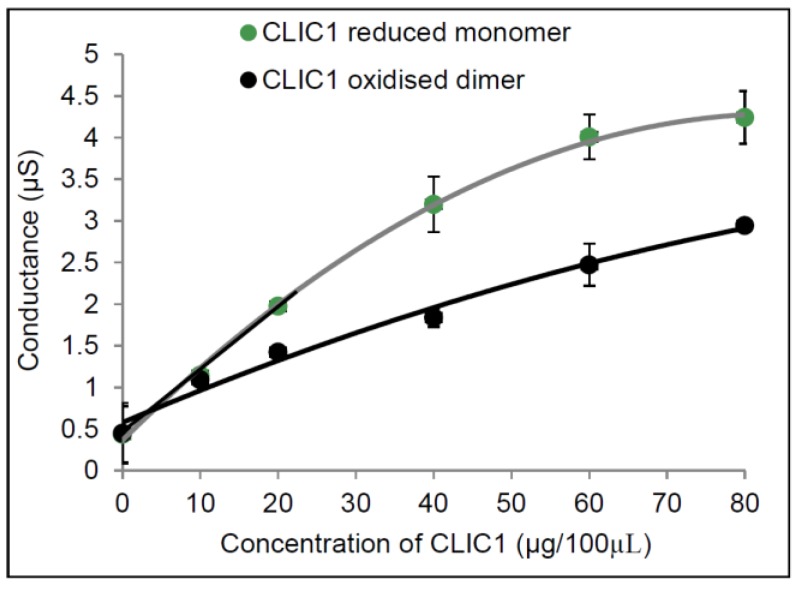
Conductance of different concentrations of CLIC1 in tBLMs containing 25 mol % cholesterol. Concentrations of 0, 10, 20, 40 and 60 µg of CLIC1 reduced monomer (pre-incubated with 0.5 mM TCEP) and CLIC1 oxidised dimer (pre-incubated with H_2_O_2_) in 100 µL of HEPES/KCl buffer (pH 6.5) were added into membranes containing 25 mol % cholesterol where the conductance was measured and linear fitting (as indicated in black for CLIC1 reduced monomer and red for oxidised dimeric CLIC1) and quadratic polynomial fits were generated using Microsoft Excel 2010 (*y* = −0.0005*x*^2^ + 0.0924*x* + 0.3639, *R*^2^ = 0.9985 for CLIC1 monomer and *y* = −0.0001*x*^2^ + 0.0399*x* + 0.5761, *R*^2^ = 0.9857). The error bars represent the standard error of three experimental repeats (*n* = 3).

In Figure 2, monomeric CLIC1 is seen to have a more linear relationship between it’s conductance versus protein concentration. At lower concentrations (10 µg and 20 µg) CLIC1 reduced monomer conduction rate is steeper (slope is 0.0769 µS/s and *R*^2^ of 0.9978) compared to CLIC1 dimer (slope = 0.0487 µS/s and *R*^2^ of 0.9664). This would suggest that at lower concentrations the CLIC1 reduced monomer form can more readily enter the membrane and/or oligomerise to form ion conductive channels in artificial membranes containing cholesterol. Of note, is that the data was fitted using a Gm vs. [CLIC]^1/2^. This suggests a model for the conduction that may arise via a mechanism other than that of an assembled multimeric ion channel. Instead an alternate model suggests that the protein interacts with the membrane via a “detergent-like” action that modifies the diameter of pre-existing toroidal pore ion pathways across the tBLM. Confirmation of this however requires further investigation and is beyond the scope of the current study.

Based on our current results and applying the knowledge we have of the distinct structural conformations adopted by the oxidised and reduced forms of CLIC1 [5], we speculate that the slower conduction rate of the CLIC1 oxidised dimer compared to reduced monomeric CLIC1, is due to its dimer form that is stabilized by an intramolecular disulphide bond formed between Cys24 and Cys59. It is postulated that the transmembrane form of CLIC1 would resemble an oxidized version of the protein, where the CLIC1 reduced form first undergoes a shape change exposing a large hydrophobic surface, that facilitates insertion into the membrane, as previously also suggested by Goodchild et al., 2009 [23]. In the absence of a membrane, the oxidized protein forms reversible non-covalent homodimers via its exposed hydrophobic surface. Our results suggest that the slower rate of conductance by oxidised dimeric CLIC1 compared to the reduced monomeric CLIC1 is due to the fact that when in the dimer form, these hydrophobic surfaces are masked and less likely to interact with the membrane. Of note, our experiments were performed in air thus it is expected that oxidation of the reduced protein would occur over time.

**Figure 3:**
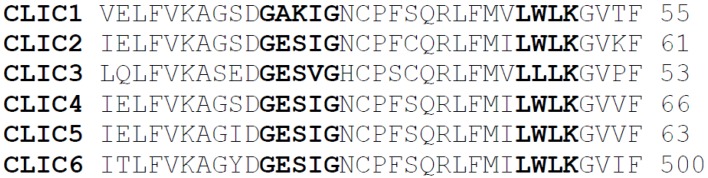
Amino Acid Sequence Alignment of Human CLIC proteins showing the CRAC motif. Highlighted in red is the GXXXG motif and in Green highlighted the LWLK motif in human CLICs. CLIC1 (accession number: CAG46868.1), CLIC2 (accession number: CAA03948.1), CLIC3 (accession number: NP_004660.2), CLIC4 (accession number: CAG38532.1), CLIC5 (accession number: AAF66928.1), CLIC6 (accession number: NP_444507.1). The alignment was produced using Clustalw.

Investigations into why certain proteins tend to associate with membranes containing higher cholesterol concentration, indicate the involvement of specific segments or motifs within the proteins themselves that facilitate interactions with specific membrane components such as cholesterol at the membrane interface [24]. For example, the interaction of the scaffolding protein flotillin and caveolins with cholesterol rich domains in membranes [24]. The cholesterol recognition amino acid consensus (CRAC) motif is located near the trans-membrane helix of some proteins and is represented by the amino acid sequence **L/V**XXXXX**R/K** or **Y**XXXXX**R/K**, where the X represents any amino acid [13]. Another CRAC segment that has a major role in sequestering proteins into cholesterol rich domains, is the tryptophan residue motif found in the fusgenic protein of HIV glycophorin-41 or gp41, represented by the sequence **LWYIK** [25]. When the Leucine residue was substituted with Isoleucine, the interaction of protein with cholesterol was found to be fully supressed. Replacement of Leucine with Alanine or Valine, resulted in both mutants having weak cholesterol binding compared to wild type protein [26]. Most human CLIC proteins not only contain a GXXXG motif, previously speculated to be involved as the cholesterol binding site [3], but they also (except for CLIC3) contain the conserved motif **L35**WLKG adjacent to their PTMD domain (Figure 3). Further investigations are required however, in order to confirm the contribution of these motifs to the changes in conductance of CLIC1 in membranes containing cholesterol.

### 2.2. Defining the Role of Redox Sensitive Residues within CLIC1

**Figure 4:**
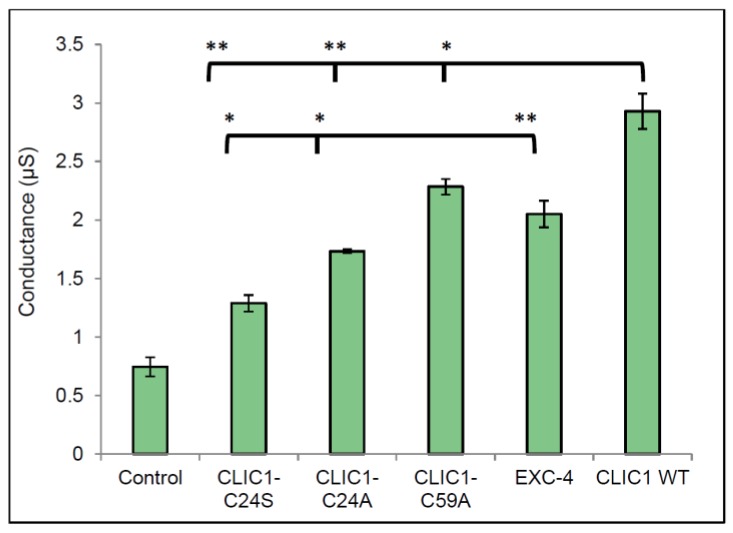
Conductance of CLIC1 mutants and EXC-4 in membranes containing 25 mol % cholesterol. 20 µg of CLIC1-C24A; CLIC1-C24S; CLIC1-C59A; EXC-4 and CLIC1 (WT) proteins in 100 µL HEPES/KCl buffer (pH 6.5) were reconstituted in tethered bilayer membranes containing 25 mol % cholesterol and the conductance was measured and analysis was performed using excel 2010 and Graph pad prism 6. Control sample is buffer only containing 0.5 mM TCEP with no protein added to membrane containing 25 mol % cholesterol. The error bars represent the standard error of three independent repeats of conductance measurements (*n* = 3).

CLIC1 contains a total of six cysteine residues, with Cys24 and Cys59 known to form an intramolecular disulphide bridge upon oxidation of the protein. CLIC1 is also the only member of the CLIC family to contain Cys59. Experiments measuring the conductance of CLIC1 mutants (CLIC1-C24A and CLIC1-C59A) were performed to determine the role of redox in CLIC1 activity. In addition, the CLIC-like protein EXC-4 was also used and compared to wild type CLIC1. In Figure 4, all proteins assessed, including the three CLIC mutants and EXC-4 were more conductive in membranes containing cholesterol, compared to the control (25 mol % cholesterol in membrane, with no protein added). Statistical analysis comparing conductance of CLIC1 wild type monomer to all CLIC1 mutants revealed a significant difference in the conductance of the tested proteins as indicated by one way ANOVA test with a *p* value of <0.0001. Further statistical analyses were performed using the Tukey’s multiple comparisons statistical test. This revealed no significant difference in conductance between the two CLIC1 mutants CLIC1-C24A and CLIC1-C24S. Similarly, CLIC1 wild type monomer and EXC-4 conductance values were not significantly different as assessed by Student *t*-test.

Structural studies of the soluble form of the CLIC proteins have demonstrated that they are members of the Glutathione-S-Transferase (GST) fold family of proteins and contain a monothiol, single cysteine redox active site [27]. We recently demonstrated that members of the CLIC family have oxidoreductase activity in their soluble form and that Cys24 in CLIC1 was critical for this enzymatic activity [28]. Due to the presence and conservation of this active cysteine residue in the structure of all human CLIC proteins (Cys24 in CLIC1), the activity of the CLICs is redox sensitive [5,28]. Experiments investigating the activity of CLIC proteins within lipid bilayers have shown that the ion channel activity of CLIC proteins in membranes is also dependent upon redox processes [5,23]. Mutation of the residue Cys24 to alanine in CLIC1 resulted in a reduction of its single ion channel conductance, compared to the wild type protein [10]. However when Cys24 was replaced by a serine residue, the ion channel activity of the protein was completely eliminated [5]. This same study also showed a complete abolition of channel activity for the mutant C59S [5].

In the current study using the two CLIC1 mutants, C24S and C24A, we found that both mutants conduct equally well in tBLMs containing cholesterol. All three CLIC1 mutants (C24A, C24S and C59A) were found to have lower conductance levels compared to wild type CLIC1 protein in membranes containing 25 mol % cholesterol. This suggests that the protein likely adopts a transmembrane structure which does not depend upon the formation of a disulphide bond between Cys24 and Cys59. The lower activity noted for both Cys24 mutants compared to WT CLIC1, supports previous findings of reduced or abolished activity for C24 mutants [5,10]. It also supports structural studies that place the Cys24 residue at the start of the putative transmembrane domain of the protein, which is predicted to span residues 24–46 [5,10]. Hence, it is not unexpected that mutation of this critical residue would impact channel conductance and/or gating. Similarly, the CLIC-like protein EXC-4 from *C. elegans*, contains an aspartic acid residue at the equivalent position to Cys24 found in CLIC1. As seen in Figure 4, Exc4 also formed functional ion channels in the tBLMs containing 25 mol % cholesterol. Under the specific conditions used for this study, it appears that redox may not be involved in regulating CLIC1 conductance or its activity once located within the membrane. There is clearly a need to further investigate redox control of CLIC1 and to determine the role of other critical residues lining the pore and the transmembrane domain of the channel.

### 2.3. Regulation of CLIC1 Conductance by Sterols in tBLMs

**Figure 5:**
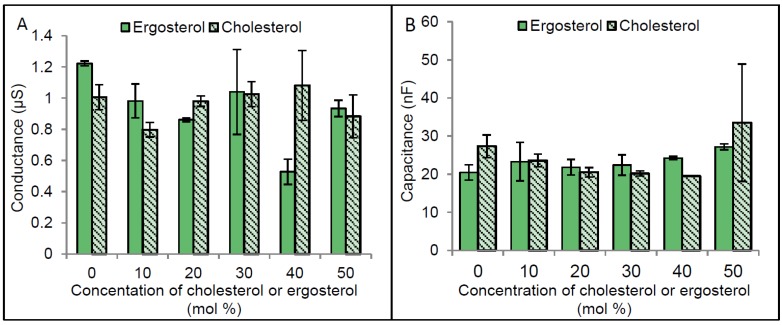
Conductance and capacitance of membranes containing different concentrations of cholesterol or ergosterol. Bilayer lipid membranes were formed using 10% tethered lipids on a gold electrode representing the monolayer of membrane and zwitterionic lipids dissolved in ethanol containing different concentrations of cholesterol or ergosterol was used as the second layer for the membrane. Membranes were then rapidly flushed with HEPES/KCl buffer (pH 6.5) in order to remove the ethanol by solvent exchange method and enhance the formation of the bilayer lipid membranes. The conductance and the capacitance of membranes were measured using impedance spectroscopy. (**A**) Represents membrane conductance at different cholesterol or ergosterol concentrations and (**B**) Capacitance of membrane with different cholesterol or ergosterol concentrations. The error bars represent the standard error of three independent impedance spectroscopy conductance measurements.

Given that the optimal functioning of a number of membrane proteins including CLIC1, have been shown to be dependent upon the presence of particular membrane lipids and specifically sterols, it was important to further explore this as a regulatory mechanism for CLIC1 channel activity. tBLMs made with varying amounts of cholesterol or ergosterol (ranging between 0 mol % and 50 mol %) were initially characterised. As seen in Figure 5, there were no significant changes to the tBLMs’ conductance or capacitance, made using a range of sterol concentrations. Statistical analysis (using two way ANOVA followed by Benferroni’s multiple comparison test) for the impedance spectroscopy measurements of membranes containing cholesterol or ergosterol, confirmed this.

Interestingly membranes containing higher ergosterol concentration become slightly better sealed (Figure 5A). Although these differences were not found to be statistically significant, they do support previous findings showing membranes containing ergosterol to be more rigid and tighly packed compared to cholesterol containing membranes [19]. Also it was previously reported that cholesterol increases the acyl chain order of phospholipids and therefore results in an increased thickness of membranes. Our capacitance measurements of membranes containing 0 to 50 mol % cholesterol or ergosterol, revealled no significant differences in membrane thickness (Figure 5B).

Published studies by others have shown that cholesterol concentrations higher than 20 mol % cause an increased disorder of artificial membranes made from PC lipids [29]. This could help explain some of the apparent fluctuations in our results. Of note is the low conductance value at 40 mol % cholesterol, which is likely anomalous, given that 30 and 50 mol % are similar. As such, a final concentration of between 20 and 25 mol % of cholesterol or ergosterol was routinely employed in this study. This level also correlates with the physiological sterol content found in most animal cells [30].

**Figure 6:**
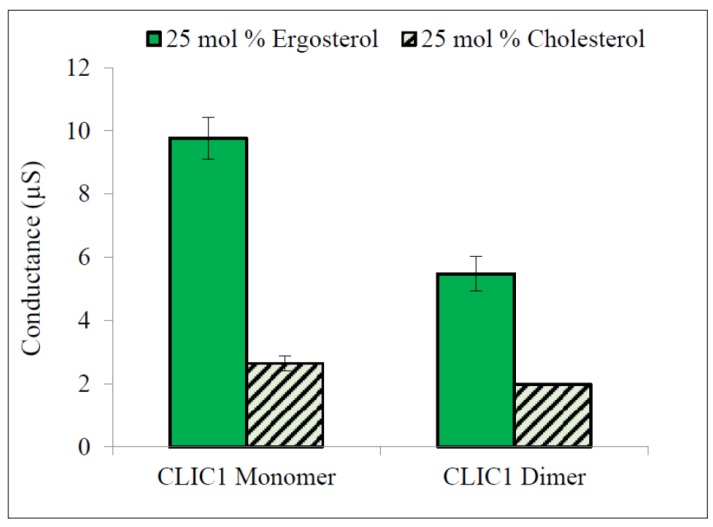
Conductance of CLIC1 in tBLMs containing 25 mol % ergosterol. 20 µg of CLIC1 monomer (pre-incubated with 0.5 mM TCEP) and dimer (pre-incubated with 2 mM H_2_O_2_) in 100 µL of HEPES/KCl buffer (pH 6.5) were incorporated into membranes containing zwitterionic lipids and 25 mol % ergosterol. The error bars represent the standard error of three independent experimental repeats (*n* = 3).

The results in Figure 6 show that both rCLIC1 monomer and rCLIC1 dimer were surprisingly greatly more conductive when added to tBLMs containing 25 mol % ergosterol compared to tBLMs containing equivalent levels of cholesterol. When quantified this translated a 3.7 fold increase in conductance for CLIC1 monomer and 2.8 fold increase in conductance for CLIC1 dimer when incorporated into tBLMs containing 25 mol % ergosterol, compared to membranes containing 25 mol % cholesterol. It has been demonstrated that at temperatures higher than 15 °C the transition of POPC membranes into the liquid ordered phase occurs at lower concentrations of ergosterol than cholesterol and indicates that ergosterol may be a more effective promoter of raft-like domains in POPC membrane compared to cholesterol [31,32]. This raises the possibility that sterol-raft domains in membranes aid in the initial binding of CLIC1 to the membrane, which likely involves the structural unfolding of CLIC1. Furthermore, sterols in membranes may play a role in the oligomerisation and final quaternary structure of the membrane protein configuration.

The stability of channels in membranes is also an important factor in maintaining the rate of ion transport across membranes [19,33]; this further suggests that the high conductance of CLIC1 in membranes with ergosterol is due to the ability of ergosterol to increase the stability of channels formed by CLIC1 via increasing the rigidity of protein aggregate structures. This may also suggest that CLIC1 monomeric protein aggregates experience a higher rigidity in the presence of ergosterol than in cholesterol membranes and therefore CLIC1 showed higher conductance with faster initiation rates in membranes with ergosterol than membranes containing cholesterol.

**Figure 7:**
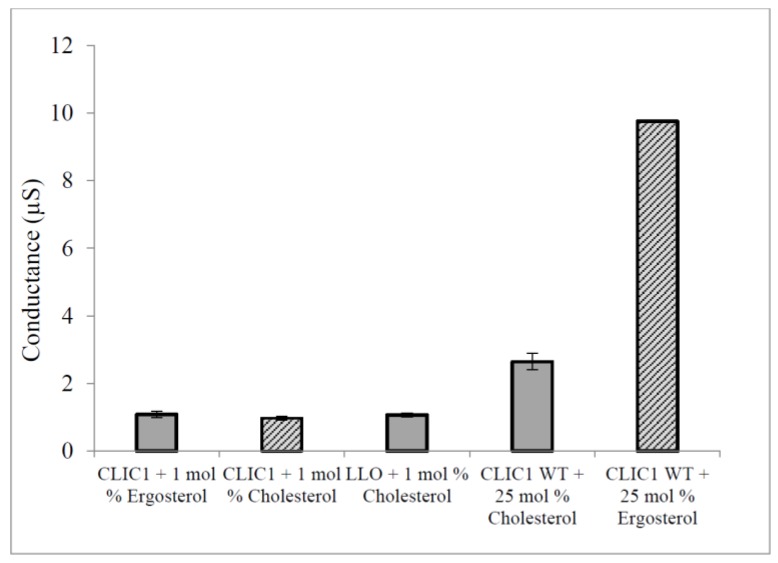
Conduction of pre-incubated CLIC1 monomer with sterols in tBLMs containing 50 mol % cholesterol or ergosterol. CLIC1 (WT) monomeric protein (20 µg in 100 µL of HEPES/KCl buffer, pH 6.5) was incubated with 1% cholesterol or 1% ergosterol for ~1 h prior addition to tethered bilayer lipid membranes containing 25 mol % cholesterol or ergosterol. Conductance of pre-incubated CLIC1 monomer with sterols was then measured with impedance spectroscopy and compared to Controls: CLIC1 monomer not pre-incubated with sterols added into membranes containing 1% of cholesterol or ergosterol, listeriolysin-O (20 µg of LLO in 100 µL of HEPES/KCl buffer, pH 6.5) was also incubated with 1% cholesterol under the same conditions as CLIC1 monomer followed by addition to membranes with 50 mol % cholesterol. The error bars represent the standard error of three repeats of experimental measures (*n* = 3).

Others have published cell based studies investigating the interaction between proteins and sterols in membranes. These were performed via hemolysis assays where Cholesterol Dependant Cytolysins (CDC) pore forming proteins were incubated with free cholesterol prior to addition to the cells. The result was an inhibition of the cytolytic activity of the toxins against erythrocytes [34,35]. Using a similar experimental set-up, CLIC1 was pre-incubated with 1% free-cholesterol or ergosterol prior to addition to tBLMs containing 50 mol % cholesterol or ergosterol. CLIC1 monomer and the CDC Listeriolysin-O (LLO) that were each pre-incubated with cholesterol, showed low conductance levels as expected (Figure 7). Interestingly, CLIC1 monomer that was pre-incubated with 1% ergosterol also showed low conductance when compared to CLIC1 monomer not pre-incubated with ergosterol or cholesterol in membranes containing 50 mol % cholesterol or ergosterol (Figure 7). The pre-incubation of CLIC1 with cholesterol or ergosterol resulting in inhibition of chloride ion channel activity in membranes, likely occurs by the sterol preventing either the initial binding and/or insertion of the protein onto the membrane, and/or its oligomerisation once located within the membrane. This strongly suggests that CLIC1 membrane interaction proceeds with initial binding to the membrane via cholesterol, which acts as a receptor or docking site for the protein, followed by oligomerisation and full assembly into functional ion channels.

In conclusion, our model tBLMs combined with impedance spectroscopy have allowed us to probe the role of both sterol and redox environment as regulators of CLIC1 spontaneous membrane insertion and ion channel activity. The high sterol-dependent conductance of CLIC1 in tBLMs strongly points to cholesterol as a receptor or initial membrane binding site for CLIC1, that may also aid in the protein’s unfolding, oligomerisation and formation of functional ion channels in membranes. Similarly, the effects of redox on CLIC1 transition between its soluble to membrane form suggests the protein first adopts a structure that likely resembles a version of its oxidized protein state, exposing a large hydrophobic surface that facilitates its membrane interaction. The redox reactive residue Cys24 located at the start of the putative TMD in CLIC1 appears not to be essential for its ion channel activity, but does influence the channel’s conductance and potentially its gating properties.

## 3. Materials and Methods

### 3.1. Preparation of His-Tagged Recombinant CLIC1 WT, CLIC1-C24A and C59A Proteins

The following annotations will be used to refer to each mutant, with each containing a single amino acid substitution (CLIC1-C24A, CLIC1-C24S and CLIC1-C59A).

Protein purification was performed as previously described [23,36]. Briefly, *E. coli* bacterial cells, BL21 (DE3) containing the His-tag pET28a vector system were grown in 2xYT media containing 50 μg/mL Kanamycin antibiotic (Sigma Aldrich, Carlsbad, Carlifonia, USA). Cells then were induced with 1 mM IPTG (Sigma Aldrich) and left to grow further at 20 °C for about 16 h with shaking at 200 rpm. Then the cells were harvested and lysed using Sonication on ice for 6 times with 10 s pulses at 80% output. Cell lysate was then ran through His-tag Ni-NTA high affinity chromatography column in the presence of 0.5 mM TCEP and the His-tagged protein was cleaved off the resin by overnight incubation with 30 NIH units of bovine plasma thrombin (Sigma Aldrich) per litre of cell culture. The eluted recombinant CLIC1 protein from the Ni-NTA resin was again incubated with 0.5 mM TCEP and ran through size exclusion chromatography using Superdex-75 prep grade high performance chromatography column (GE healthcare, Piscataway, NJ, USA) in order to obtain 99% purity of monomeric protein. The chromatography column was initially equilibrated in column sizing buffer with reducing agent (100 mM KCl, 0.5 mM TCEP, 1 mM NaN_3_, and 20 mM HEPES pH 7.5) and the purification was performed at 4 °C. The purified proteins were quantified using the BCA protein assay (Thermo Scientific, Sydney, Australia) and the purity and oligomeric state of proteins was further investigated by running SDS-PAGE.

### 3.2. Recombinant CLIC1 Dimeric Protein

Dimeric CLIC1 was provided by Dr Louise Brown from Macquarie University, Australia. It was prepared as previously described [23,36].

### 3.3. Preparation of Recombinant EXC-4 and CLIC1-C24S by GST Gene Fusion System

Glutathione S-Transferase (GST) Gene fusion system (AMRAD-Pharmacia, Melbourne, Australia) was used for the expression and purification of fusion proteins in *E. coli* bacteria. *E. coli* bacteria strain, BL21 (DE3) containing pGEX-4T-1 vector (Novagen, ON, Canada) was left to grow in 2xYT media containing 100 µg/mL Carbenicillin on a shaker at 180 rpm, at 37 °C for 2.5 h or until an OD of 600 was achieved. Cells then were induced with 1 mM IPTG and returned to incubation for another 4.5 h at 37 °C with 180 rpm shaking. Cells were harvested and lysed by sonication as described in the previous section. Then purification of the cells lysate was achieved by running it through the glutathione-sepharose 4B resin (Amersham Biosciences, Sydney, Australia) in the presence of 0.5 mM TCEP where the GST-tagged proteins were cleaved off from the resin beads by incubation with 30 NIH units per 1 L of cells culture of bovine plasma thrombin (Sigma Aldrich) as described in the section above. Then the recombinant proteins were further purified by size exclusion chromatography in the presence of 0.5 mM TCEP as the reducing agent in order to obtain reduced monomeric proteins with size exclusion chromatography profile containing one peak. The protein concentration and purity was determined as was described in the section above.

### 3.4. Formation of Tethered Bilayer Lipid Membranes (tBLM)

Artificial membranes of ~4 nm thickness capable of incorporating proteins of up to 40 kDa were formed using methods reported in [3,22,37,38]. The monolayer tethering coating was prepared by coating freshly deposited, 100 nm pattern gold electrodes, on 25 mm × 75 mm × 1 mm polycarbonate slides, with two benzyl disulphide families, one being a spacer molecule containing a four oxygen-ethylene glycol spacer, terminated with an OH group (90%), and the second being a tethering group comprising an eleven oxygen–ethylene glycol linker group with a single C20 hydrophobic phytanyl chain (10%) as the hydrophobic tether. The gold electrode was then assembled onto a 6 well polyethylene cartridge, possessing a 2 mm^2^ active area and a flow cell chamber of 100 μm in height. 8 µL of 3 mM mobile lipid phase (MLP) containing 70% zwitterionic C20 diphytanyl-ether-glycero-phosphatidylcholine: 30% C20 diphytanyl-diglyceride ether lipids dissolved in 99% (*v*/*v*) ethanol was added into each of the 6 wells and allowed to incubate at room temperature for ~2 min. For making the second layer of membranes, cholesterol or ergosterol (both from Sigma Aldrich) that were dissolved in 95% (*v*/*v*) ethanol were mixed with the MLPs to make different concentrations (0 mol %–50 mol %). This process was then followed by rinsing the lipids with 3 × 100 µL of HEPES/KCl buffer: 0.1M KCl, 0.1 mM HEPES and 0.01 mM CaCl_2_, pH 6.5. Rinsing away the ethanol solvent and replacement with an aqueous buffer drives the rearrangement of the dissolved lipids to form a lipid bilayer, which is detected by the changes in impedance spectroscopy measurements.

### 3.5. Formation of tBLM Using Yeast and Bacterial Lipids

Lipid extracts of yeast (*Saccharomyces cerevisiae* from BioAustralis Pty Ltd., Sydney, Australia) and *E. coli* bacterial cells (provided by Dr Charles Cranfield from the Victor Chang Institute for Medical Research, Sydney, Australia), were dissolved separately in 95% (*v*/*v*) ethanol with the aid of heating in a 50 °C water bath followed by continuous vortexing for at least 15 min. 3 mM of yeast or *E. coli* lipid solution was added to the first layer of membrane in the coated gold electrode in place of the mobile lipid solution and tBLM formation, as described in the section above.

### 3.6. Incorporation of CLIC1 WT, Mutants and EXC-4 into tBLMs Containing Cholesterol

Recombinant CLIC1 (WT) monomeric and dimeric proteins; CLIC1-C24A; CLIC1-C59A and EXC-4 (WT) were diluted to a concentration of 20 µg/100 µL (7.4 µM) in HEPES/KCl buffer (0.1 M KCl, 0.1 mM HEPES and 0.01 mM CaCl_2_ of pH 6.5). Each protein was incubated with 0.5 mM TCEP for ~1 h. They were then applied to pre-prepared tethered bilayer lipid membranes with or without cholesterol or ergosterol that were equilibrated for ~1 h with 100 µL of HEPES/KCl buffer containing 0.5 mM TCEP when measuring the conductance of monomeric reduced CLIC1 or membranes were equilibrated with 2 mM H_2_O_2_ when conducting experiments with oxidized or dimeric CLIC1.

### 3.7. Pre-Incubation of CLIC1 with Cholesterol or Ergosterol

CLIC1 (WT) monomeric protein (20 µg in 100 µL of HEPES/KCl buffer) was incubated for approximately 1 h with 2 µL of 13.3 mg of cholesterol or ergosterol dissolved in 1 mL of 95% (*v*/*v*) ethanol prior addition to tBLMs with or without sterols.

### 3.8. Pre-Incubation of Listeriolysin-O with Cholesterol

Listeriolysin-O (Sigma Aldrich), 2 µM in 100 µL as a final volume of HEPES/KCl buffer was incubated with 2 µL of 13.3 mg of cholesterol dissolved in 95% (*v*/*v*) ethanol before application to membranes with or without cholesterol.

## 4. Conclusions

This study has employed an artificial tethered lipid bilayer system to demonstrate the presence of sterols (cholesterol or ergosterol) within the bilayer is critical for the ion channel conductance activity of the protein CLIC1. Furthermore, the oxidation state of the protein also serves to regulate its channel conductance activity. While the redox active residue Cysteine 24 in CLIC1 although essential for its oxidoreductase enzymatic activity, does not appear to be essential for its ion channel conductance activity.
